# Serotonin Syndrome-Mimicking Manifestations in a Patient with Systemic Lupus Erythematosus

**DOI:** 10.3390/jcm13123516

**Published:** 2024-06-15

**Authors:** Shih-Chi Chen, Yan-Siang Huang, Chien-Sheng Wu

**Affiliations:** 1 Department of Internal Medicine, Far Eastern Memorial Hospital, New Taipei City 22060, Taiwan; jianpi2004@gmail.com; 2 Department of Neurology, Far Eastern Memorial Hospital, New Taipei City 22060, Taiwan; huang.eason@gmail.com

**Keywords:** neuropsychiatric systemic lupus erythematosus, serotonin syndrome, anti-ribosomal P antibody, magnetic resonance imaging

## Abstract

Neuropsychiatric systemic lupus erythematosus (NPSLE) is a complication of systemic lupus erythematosus with diverse clinical presentations sharing common features with variable neurologic disorders. Magnetic resonance imaging (MRI) may provide imaging evidence of structural brain abnormalities associated with symptoms of NPSLE. Serotonin syndrome is a toxidrome characterized by altered mental status, autonomic hyperactivity, and neuromuscular abnormalities. It is mostly caused by medications that increase serotonin and is rarely reported as a manifestation of neuropsychiatric lupus. We presented the case of a 24-year-old Taiwanese woman with a history of systemic lupus erythematosus diagnosed at 21 years of age. The initial clinical and laboratory presentations upon diagnosis included fever, arthritis, hypocomplementemia, positive antinuclear antibody, anti-double-stranded DNA antibody, and anti-ribosomal P antibody. Her condition once remained stable under oral glucocorticoids and immunosuppressants, but she developed sudden-onset consciousness disturbance, incoherent speech, and unsteady gait ten days before our assessment. A high fever of up to 39 °C with tremor and clonus occurred at the intensive care unit. Brain MRI revealed symmetric T2 hyperintensity without diffusion restriction over the bilateral globus pallidus. High-dose pulse glucocorticoid and rituximab were prescribed during her admission and the neuropsychiatric symptoms diminished upon treatment. No alternation in mental status or involuntary movements were noted at follow-up. Our patient was diagnosed with neuropsychiatric lupus, with clinical symptoms and image findings mimicking those of serotonin syndrome. Neuroimaging, such as MRI, detects various structural brain abnormalities and may provide pathophysiological evidence of clinical manifestations.

## 1. Introduction

Systemic lupus erythematosus (SLE) is a chronic autoimmune disorder characterized by multisystem involvement and a broad spectrum of autoantibodies. Neuropsychiatric symptoms of SLE are highly variable; the diagnosis relies heavily on clinical judgment since no single laboratory or imaging study is sensitive or specific enough for definite confirmation [1]. The association between anti-ribosomal P antibody and the presence of neuropsychiatric lupus (NPSLE) has been well-established, but the pathogenic role of the autoantibody remains unclear [2,3,4]. Neuroimage protocols of magnetic resonance image (MRI) are used extensively to define the anatomic basis of NPSLE [5,6,7,8].

Serotonin syndrome (SS) is a potentially life-threatening condition caused by increased serotonergic activity in the central nervous system [9]. It is often precipitated using serotonergic drugs and is considered a severe drug reaction. Classical symptoms include altered mental status, such as agitation and cognitive impairment, autonomic hyperactivity, such as hyperthermia and hypertonia, and neuromuscular abnormality, such as clonus, though only a proportion of patients present with all the findings. Bilateral globus pallial lesions are a characteristic finding of serotonin syndrome on MRI [10,11].

We here report a systemic lupus erythematosus patient with a positive anti-ribosomal P antibody, suffering from neuropsychiatric lupus with serotonin syndrome-mimicking clinical manifestations. MRI of the brain was performed during her acute confusion status and revealed bilateral globus pallidus lesions, a finding correlated with serotonin syndrome.

## 2. Case Report

### 2.1. Patient Presentation

A 24-year-old Taiwanese woman was diagnosed with SLE at age 21, with initial presentations of fever, fatigue, myalgia, joint pain, and malar rash. Laboratory studies performed at the outpatient clinic revealed hypocomplementemia, positive antinuclear antibody, anti-double-stranded DNA antibody, and anti-ribosomal P antibody. She began receiving treatment, and the previous symptoms improved under the use of prednisone, hydroxychloroquine, and cyclosporine. No signs of disease flare-up were reported.

After three years of regular follow-up, she developed a sudden onset of altered mental status. According to her mother, the patient became drowsy during daytime and anxious at night, and insomnia with frequent nightmares was also complained of. Incoherent speech, unsteady gait, and hallucinations (bugs crawling inside the head, as referred by the patient) took place for one week, so she was admitted to the emergency department of our hospital.

### 2.2. Physical, Laboratory, and Imaging Findings upon Admission

At the triage, she presented with a temperature of 38.9 °C, heart rate of 121 beats per minute, respiratory rate of 14 per minute, blood pressure of 132/76 mmHg, and peripheral oxygen saturation of 97% under ambient air. Her Glasgow Coma Scale (GCS) was E4M6V5, but she was disoriented to time and place. A preliminary neurologic examination showed normal muscle power and sensation, and no cranial nerve defects were present. Chest roentgenography and urine analysis revealed no evidence of infection. Intravenous methylprednisolone 20 mg per 12 h was also given.

### 2.3. Clinical Course after Hospitalization

A rapid deterioration of consciousness took place on the third day of her stay at the emergency department. The vital signs were relatively stable (temperature of 37.1 °C, heart rate of 96 beats per minute, respiratory rate of 12 per minute, and blood pressure of 139/88 mmHg), but the GCS went downhill to E3V1M3, so she was intubated and sent to the intensive care unit (ICU). Laboratory tests revealed anti-dsDNA antibody > 444 IU/mL, C3 48.9 mg/dL, C4 < 8 mg/dL, anti-cardiolipin antibody 14.0 GPL-U/mL, and anti-ribosomal P antibody 232 EU/mL. A lumbar puncture was performed, the open pressure of cerebrospinal fluid (CSF) was 108 mmH_2_O, and the close pressure was 90 mmH_2_O, with no white blood cells seen under a high-power field. 

Intravenous ceftriaxone 2000 mg per 12 h was prescribed empirically for possible CNS infection, though the CSF culture eventually yielded negative results. Methylprednisolone 20 mg per 12 h was continued along with hydroxychloroquine and cyclosporine for the underlying SLE. She regained consciousness two days after arriving at the ICU, and she was extubated smoothly. However, an episode of fever up to 39 °C recurred the day after, along with hypertension up to 170/96 mmHg, hyperventilation of respiratory rate 20 per minute, and agitated mood and behaviors. She then developed limb tremor and clonus. These presentations resemble serotonin syndrome, so biperiden, diphenhydramine, and antiepileptic agents were given, though her family’s statement and previous medical records showed no history of exposure to serotonin agents.

**Figure 1:**
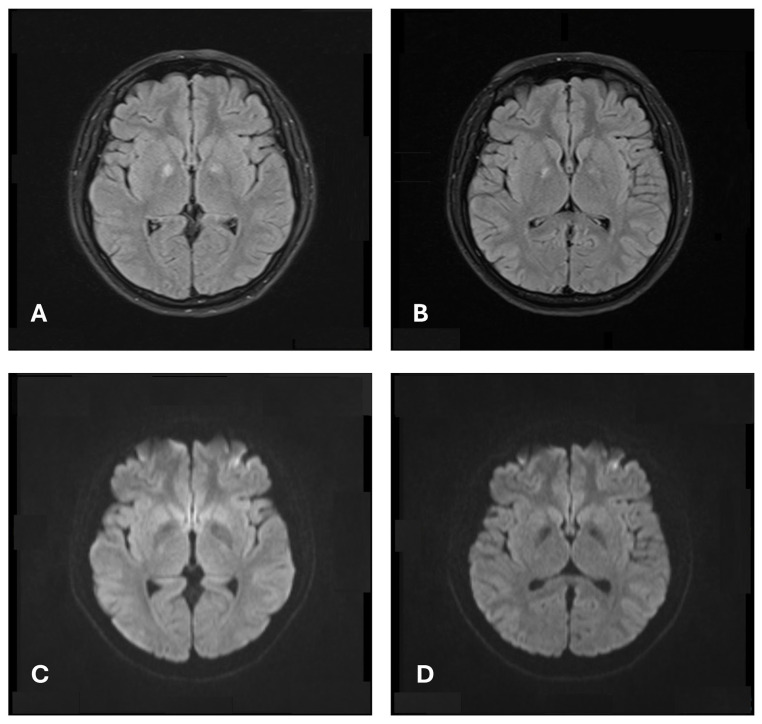
Brain magnetic resonance imaging. Axial T2-weighted fluid-attenuated inverse recovery images showed bilateral globus pallidi hyperintensity (**A**) without diffusion restriction in diffusion-weighted image (DWI) (**C**). Follow-up MRI showed partially regression of globus pallidial lesions (**B**) without diffusion restriction in diffusion-weighted image (**D**).

A further electroencephalography (EEG) study reported excessive beta activity without evidence of non-convulsion seizure or epileptogenicity. MRI of the brain revealed symmetric T2 hyperintense bilateral globus pallidus lesions without diffusion restriction (Figure 1A,C), a finding noted in metabolic or hypoxic encephalopathy caused by carbon monoxide (CO) poisoning, cocaine overdose, or serotonin syndrome. These conditions were all carefully ruled out by history-taking and laboratory examinations.

### 2.4. Treatment and Outcomes

Meropenem and acyclovir were given empirically for intensive coverage of CNS infection. A three-day course of pulse corticosteroid therapy with methylprednisolone 1000 mg was also given from the following day of the clonus attack. The fever subsided within 48 h so both anti-bacterial and anti-viral agents were discontinued after a 7-day course. Her consciousness and mood gradually stabilized after completing pulse corticosteroid therapy, and she was then transferred to the general ward on the seventh day of admission. No further clonus recurred, but psychiatric symptoms such as anxiety and hallucinations were complained of. A second course of pulse corticosteroid therapy with methylprednisolone 1000 mg was repeated from the 10th to the 12th day of admission, followed by rituximab 500 mg on the 13th day. Her consciousness level and mental status finally returned to baseline, and the titer of anti-ribosomal P antibody decreased to 92 EU/mL. She was discharged after a 14-day admission course. A follow-up brain MRI was performed ten months later, showing partially resolved bilateral pallidal lesions without other remarkable findings (Figure 1B,D). No residual or recurrent motor symptoms were present thereafter, but she still felt some anxiety during her latest clinical visit.

## 3. Discussion

Our patient presented with fever, altered mental status, and clonus during her admission, as well as bilateral global pallidal lesions on MRI, which resemble serotonin syndrome. Serotonin syndrome is a disorder induced by pharmacologic treatment with serotonergic agents that increase serotonin activity [9]. The largest population of serotonergic neurons is located in the midbrain and extends to the basal ganglia [12], so symptoms related to basal ganglia injuries might mimic serotonin syndrome.

MRI of our patient during acute confusion showed hyperintense, T2-weighted, bilateral globus pallidus gray matter lesions, rather than the MRI findings of other NPSLE showing hyperintense, T2-weighted, focal white matter lesions in the brain [13,14]. Image findings like those of our patient have been reported in cases with serotonin syndrome caused by drug overdose [15]. They can also be found in CO intoxication, opiate overdose, or cyanide poisoning [10,11,16]. Patients with CO intoxication usually have an exposure history and present with distinct findings such as cherry-red skin, cyanosis, nausea, or vomiting and can be differentiated from our case by physical examination. Other differential diagnoses were ruled out by medical history, so NPSLE-induced basal ganglia injuries were strongly implied as the cause behind both clinical symptoms and image findings.

Psychiatric presentations in NPSLE, such as psychosis, anxiety, and cognitive dysfunction, may theoretically be related to basal ganglia injuries. The basal ganglia are primarily responsible for motor control, but increasing evidence has demonstrated their involvement in cognitive function. Notably, the globus pallidus, a part of basal ganglia, is usually affected in cerebral calcinosis in SLE, implying that involvement of the areas associated with serotonin syndrome may be more common that previously recognized [17]. When facing excitotoxicity or hypoxia-ischemic conditions, the metabolically active nature of the basal ganglia makes them vulnerable to damage, leading to abnormal signals shown on MRI [18].

The American College of Rheumatology subcommittee categorized NPSLE into 19 distinct syndromes, from central nervous system disorders to peripheral neuropathy [19]. Despite its broad coverage, the ACR classification still underestimated the diversity of the disease, and presentations beyond the categories have been widely reported. Diagnosing NPSLE remains challenging due to the lack of universal serum or CSF biomarkers holding clinical significance [20], but a few autoantibodies have been associated with the presence of neuropsychiatric symptoms in SLE. The anti-ribosomal-P antibody, an antibody against three phosphorylated protein components of ribosomes, is presented in 10–47% of SLE patients and is among the most studied [3]. Previous in vitro studies have shown that the anti-ribosomal P antibody induces inflammatory response by binding to T cells, monocytes, neurons, and hepatocytes [21]. Injection of the antibody into brain ventricles of rats also triggered electrophysiologic and behavioral changes [22], indicating its possible role in the development of NPSLE. The anti-ribosomal-P protein antibody is clinically related to symptoms such as psychosis or depression in SLE, but its distinct immunopathology and influence on the neuroanatomical structures in our patient are not clear [3]. Our patient, however, was found positive for anti-ribosomal P protein antibody upon the diagnosis of SLE and did develop neuropsychiatric symptoms afterward, which implied a possible pathogenic role of the antibody in the development of NPSLE.

The precise pathogenesis of NPSLE is still unclear to date, but multiple approaches have shed light on the directions of further research. The brain MRI of our patient is compatible with the functional neuroanatomy of serotonin syndrome, while a previous case report of neuropsychiatric lupus shows striatal encephalitis which resembles anti-N-methyl-D-aspartate receptor (anti-NMDAR) encephalitis involving the striatum [23]. In addition, image techniques and functional anatomy of the nervous system may be applied for the classification of NPSLE. New image analysis techniques such as self-supervised contrastive learning, volumetric measures of globus pallidus, or quantitative susceptibility mapping as well as activation pattern assessed by functional MRI may detect subtle serial changes in normal-appearing areas and provide new insight into its pathogenic pathway [6,24,25,26], which hopefully will lead to the improvement in diagnosis and management.

Despite concerns regarding their efficacy and toxicity, high-dose glucocorticoid and cyclophosphamide remain the mainstay treatment for NPSLE [27]. Rituximab, a monoclonal antibody against the B cell surface protein CD20, may be a reasonable choice for a woman of reproductive age with autoantibody-mediated manifestations of SLE, though further supportive evidence is required [28].

## 4. Conclusions

Our case demonstrated the complex yet patterned nature of neuropsychiatric symptoms in SLE. Though the patient presented rarely reported serotonin syndrome-like manifestations, they could be correlated with the image findings on MRI, implicating the effect of an injury to the basal ganglia. Disease involvement of the nervous system should always be put in mind when neuropsychiatric symptoms appear in SLE patients, especially those with a positive anti-ribosomal P antibody. Neuroimaging, such as MRI, detects various structural brain abnormalities and may provide a pathophysiology explanation for clinical manifestations.

## Data Availability

The original contributions presented in the study are included in the article, further inquiries can be directed to the corresponding author.
